# Salvation

**Published:** 1890-09-20

**Authors:** 


### Words of Consolation.

### SALVATION.

"Brethren, my heart's desire and prayer for Israel is, that they might be saved." In these words St. Paul strikes a note which thrills through the heart of every one who loves his fellow creatures. It is the most important object in the world to save souls. Unfortunately those who most require salvation are the least likely to see the necessity of it. Let us ask ourselves what is it to be saved? and in what way do we need salvation? By comparing what we do for the body with what we ought to do for our souls, we may learn. Imagine then being awoke suddenly in the dead of night, strange noises sound in our ears and around us is a stifling smoke. In a moment we realise that the house is on fire, and oh! horror, we cannot escape from destruction. We fly to the window and look out; a crowd is below, but no one can reach us, the flames are leaping up and up and ever creeping nearer and nearer. Then we cry to the Lord in our trouble, *will* He deliver us from our distress? Yes, one brave man ventures his life, and by the aid of the fire escape we are saved. Could we ever forget such a moment or be ungrateful to the hero who had ventured his life for ours? So we think, but are we grateful to the Saviour who gave His life for us, to save us from the fires of Hell?

Again, we are walking carelessly, foolishly near to the edge of a cliff overhanging the sea, which rolls and sparkles below us. Something attracts our notice, heedlessly we make a false step, or are about to do so, when a strong hand clutches us and drags us back and saves us from perishing. We struggle, perhaps, at first, angry at the rough treatment which was necessary to deliver us from peril; but soon we tremble as we realise how we might have been dashed to atoms. We know then what saving the body means; should we not be equally ready to save our souls? Christ has once for all saved mankind from all the punishment of sin. It depends on man himself whether he will take the gifts.

Going down a fire escape must be a difficult and uncomfortable thing to accomplish, but who would refuse to try it in their extremity? Our Lord says, "Follow Me in the narrow way, and do not go near temptation; it looks pleasant, but it is dangerous." Ought we not to obey His voice, and curb our ill-tempers and evil wishes? It is better to receive rough treatment, such as sorrow or sickness or adversity, from His hand, if it drags us from the edge of the bottomless pit. Let us not struggle and fight with the hand which saves us.

When the angel told the mother of our Lord that she should have a son, he added, "And thou shalt call His Name Jesus, for He shall save His people from their sins." Yes, save them from committing sin, prevent them from offending their Maker, keep them from crucifying afresh the Son of God. It would be a selfish, mean thing, only to wish to be saved from punishment; to go on indulging our evil passions to the last, and on our death-beds to expect forgiveness. Oh! shameful thoughts! To have no love nor gratitude, nor desire for a holy life in order that we may please the sinless God, no prayer that we may be saved *from* our sins! We must ask to be saved from our sins *now*; we must pray to be kept from committing fresh offences; we must deny ourselves and follow in the narrow way which leadeth to eternal life. Let us get rid of selfishness as much as we can, and make our love generous and free as was the love of our Blessed Saviour. Let us encourage such thoughts as those in the beautiful hymn:—

My God, I love Thee; not because
  I hope for heaven thereby,
Nor yet because who love Thee not
  Are lost eternally.

Not from the hope of gaining aught,
  Not seeking a reward;
But as Thyself hast loved me,
  O ever-loving Lord.

